# Bayesian Covariate-Dependent Gaussian Graphical Models with Varying Structure

**Published:** 2022

**Authors:** Yang Ni, Francesco C. Stingo, Veerabhadran Baladandayuthapani

**Affiliations:** Department of Statistics, Texas A&M University, College Station, TX 77843, USA; Department of Statistics, Computer Science, Applications “G. Parenti”, The University of Florence Florence, Italy; Department of Biostatistics, University of Michigan, Ann Arbor, MI 48109, USA

**Keywords:** Covariate-dependent graphs, Markov random fields, Random thresholding, Subject-level inference, Undirected graphs

## Abstract

We introduce Bayesian Gaussian graphical models with covariates (GGMx), a class of multivariate Gaussian distributions with covariate-dependent sparse precision matrix. We propose a general construction of a functional mapping from the covariate space to the cone of sparse positive definite matrices, which encompasses many existing graphical models for heterogeneous settings. Our methodology is based on a novel mixture prior for precision matrices with a non-local component that admits attractive theoretical and empirical properties. The flexible formulation of GGMx allows both the strength and the sparsity pattern of the precision matrix (hence the graph structure) change with the covariates. Posterior inference is carried out with a carefully designed Markov chain Monte Carlo algorithm, which ensures the positive definiteness of sparse precision matrices at any given covariates’ values. Extensive simulations and a case study in cancer genomics demonstrate the utility of the proposed model.

## 1. Introduction

Undirected Gaussian graphical models (GGMs), also known as Gaussian Markov random fields, are one of the common tools to analyze multivariate data with complex structure and find many useful applications across biomedicine, finance, and public health. A GGM can simply be expressed as a multivariate Gaussian distribution with a sparse precision (inverse-covariance) matrix. The zero entries of the precision matrix have probabilistic interpretation of conditional independence between the Gaussian random variables (nodes of a graph). Moreover, all the conditional independence relationships can be directly estimated from the accompanying undirected graph for which a zero entry in the precision matrix corresponds to a missing edge in the graph. This equivalency, essentially reduces the problem of graph structure learning in GGMs to finding zeros in the precision matrix.

Many existing GGM approaches (Dobra et al., 2004; Sudderth et al., 2004; Meinshausen and Bühlmann, 2006; Yuan and Lin, 2007; Friedman et al., 2008; Scott and Carvalho, 2008; Dobra et al., 2011; Green and Thomas, 2013; Drton and Maathuis, 2017; Khare et al., 2018; Massam, 2018; Gan et al., 2019) assume an independent and identically distributed (i.i.d.) sampling scheme ***y***_*i*_ = (*y*_*i*1_, … , *y*_*ip*_) ~ *N* (0, **Ω**^−1^) for *i* = 1, … , *n* where **Ω** is the precision matrix. However, the independence assumption does not hold in many applications. For example, observations in multivariate time series data are not independent and exhibit temporal correlations; similarly for spatial data with spatial correlation. In addition, the assumption of identical distribution implies homogeneity across observations and is often violated as well. For instance, tumor heterogeneity is a well-known characteristic in cancer: patients with the same cancer-type can be rather different in their genetic/genomic architecture. Forcing the same GGM (i.e., the same precision matrix **Ω**) onto every patient is a restrictive modeling assertion, when modeling cancer genomic networks.

Attempts have been made to extend GGMs or other types of graphical models beyond i.i.d. data. If there is a natural grouping of the observations, *multiple graphical models* (Guo et al., 2011; Danaher et al., 2014; Oates et al., 2014; Peterson et al., 2015; Yajima et al., 2015; Xie et al., 2016; Ni et al., 2018; Shaddox et al., 2018) can be applied to learn group-specific graphs assuming observations within each group are i.i.d.. Another line of work incorporates additional covariates ***x***_*i*_ in estimating graphs. *Conditional Gaussian graphical models* (Rothman et al., 2010; Yin and Li, 2011; Bhadra and Mallick, 2013) are multivariate linear regression models with the error terms following an i.i.d. GGM (can be viewed as chain graphical models). While graph estimation is conditional on the covariates, they only enter the model via the mean structure. As a consequence, the graph topology and the precision matrix stay the same across observations. In this paper, we are taking a more direct approach, in the sense that the latent graph and hence the sparse precision matrix are explicit functions of covariates.

There are a few recent work in this direction. Liu et al. (2010a) proposed a tree-based method that partitions the covariate space into a finite number of subspaces by classification and regression trees and fits GGMs separately to subsets of data. However, the estimated graphs may be unstable and lack similarity for similar covariates due to the separate graph estimation, as reported by Cheng et al. (2014). Kolar et al. (2010) proposed a penalized kernel smoothing approach that allows the precision matrix to vary with covariates. Cheng et al. (2014) developed a conditional Ising model for binary data where the dependencies are linear functions of covariates. Although the methods of Kolar et al. (2010) and Cheng et al. (2014) allow edge strength to vary with covariates, graph structure is assumed to be constant across all observations. Recently, Ni et al. (2019) proposed a graphical regression framework that allows both edge strength and graph structure to vary with covariates in (directed) Bayesian networks. They assumed there exists a natural ordering of the nodes. Given this assumption, Bayesian networks can be written as systems of recursive linear regressions. A conditional independence function was then introduced to connect regression coefficients with covariates.

**Figure 1:**
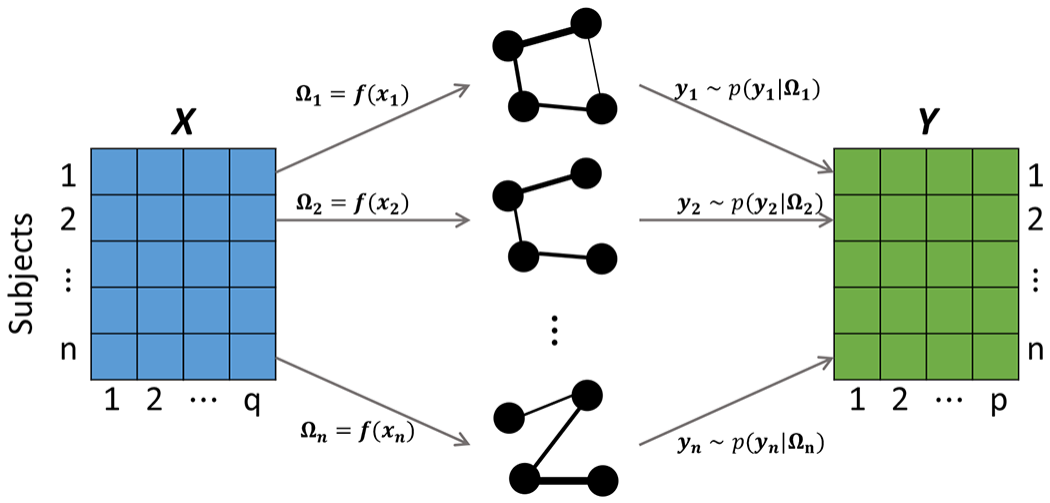
Illustration of GGMx. Subject-level sparse precision matrices **Ω**_*i*_ and graphs *G_i_* of ***Y*** vary with covariates ***X***. The edge thickness is proportional to its strength of association *ω_ijk_*. Both edge strength and graph structure change with ***X***. GGMx can also be viewed as a generative model: ***X*** generate graphs which in turn generate ***Y***.

In this paper, we consider a general problem of estimating *undirected* GGMs conditional on covariates (GGMx). GGMx allows not only the edge strength (i.e., off-diagonal elements of precision matrix) but also the graph structure (i.e., sparsity pattern of precision matrix) to vary as functions of covariates, which is illustrated in Figure 1 with graphs with four nodes and two covariates. Figure 1 also illustrates the generative mechanism underlying GGMx: covariates ***x***_*i*_ generate sparse precision matrices **Ω**_*i*_ (hence the graphs *G_i_*), which in turn generate responses ***y**_i_*. The major challenge in this context is the positive definiteness constraint of precision matrices – a *sine qua non* for GGMs – in the presence of covariates. We propose a simple strategy by specifying a matrix-valued function ***f***(·), such that **Ω**_*i*_ = ***f***(***x**_i_*) is a positive definite matrix for any ***x**_i_* *almost surely*; along with the function ***f***(·) consisting of a random thresholding component that encourages sparse precision matrix estimation, specifically enforcing the required zero-pattern that corresponds to missing edges. The sparse functional relationship between **Ω**_*i*_ and ***x**_i_* allows for novel graph interpolation for an unseen observation at covariates x*. We show that the random thresholding gives rise to a discrete mixture of *non-local priors* (Johnson and Rossell, 2010) *for precision matrices*. We also carefully design a Markov chain Monte Carlo (MCMC) algorithm for posterior inference, which guarantees to propose positive definite precision matrices for any ***x****_i_*. GGMx allows for subject-level inference on unknown graphs. Moreover, GGMx is a general class of graphical models, which subsumes at least five special cases including standard GGMs, group-specific GGMs (Guo et al., 2011; Danaher et al., 2014; Peterson et al., 2015), time-varying GGMs (Zhou et al., 2010), covariate-dependent GGMs (Kolar et al., 2010), and context-specific GGMs (Nyman et al., 2017). Extensive simulation studies show strong and robust performance of GGMx compared with competing methods. Using a cancer genomics case study, we demonstrate how GGMx can be used to infer subject-specific gene networks, which can facilitate deeper investigations in the genomic foundation of precision medicine.

The rest of this article is organized as follows. We introduce the background and notations in Section 2. We present the proposed GGMx in Section 3 and discuss the link between the random thresholding prior and non-local priors in Section 4. We summarize the posterior inference and graph interpolation in Section 5. We demonstrate the utility and robustness of GGMx with extensive simulation studies in Section 6. GGMx is illustrated by a real data application in Section 7. Section 8 provides our closing discussion.

## 2. Background and notation

A GGM is a multivariate Gaussian distribution with a sparse precision matrix. Let $Y=(Y_{1},\ldots ,Y_{p})∼N\left(0,\Omega^{-1}\right)$ be multivariate Gaussian random variables with mean zero and precision matrix $\Omega =\left[\omega_{jk}\right]$. Since the off-diagonal elements in **Ω** are proportional to partial correlations, a zero entry *ω_jk_* = 0 indicates that *Y*_*j*_ and *Y*_*k*_ are conditionally independent given all other variables. A GGM graphically represents the zero patterns of **Ω** by an undirected graph. An undirected graph *G* = (*V, E*) consists of a set of nodes *V* = {1, …, *p*} and a set of undirected edges E⊆{{j,k}∣j,k∈V}. The nodes *V* represent the variables ***Y*** and an edge {*j*, *k*} is present in the graph if and only if $\omega_{jk}\neq 0$. This is not an arbitrary way of drawing a graph. In fact, the conditional independence relationships that are encoded in the multivariate Gaussian distribution can be directly read off from *G* using the notion of graph separation. Importantly, learning the graph structure is equivalent to finding the zero patterns of **Ω**.

Under the Bayesian paradigm, several prior distributions (Roverato, 2002; Wang et al., 2012; Wang, 2015) for sparse precision matrices have been developed, which all take the same general form,

(1) $$\pi (\Omega )=\frac{\tilde{\pi }(\Omega )I\left(\Omega \in M^{+}\right)}{\int \tilde{\pi }(\Omega )I\left(\Omega \in M^{+}\right)d\Omega }\propto \tilde{\pi }(\Omega )I\left(\Omega \in M^{+}\right),$$

with *M*^+^ being the collection of positive definite matrices (PDMs). For example, G-Wishart prior (Roverato, 2002) assumes $\tilde{\pi }(\Omega )$ to be a Wishart distribution $Wishart\left(\cdot |b,\Omega_{0}\right)$ and $M^{+}≔M_{G}^{+}$ to be PDMs consistent with a graph *G*, which leads to $\pi \left(\Omega |G,b,\Omega_{0}\right)\propto Wishart\left(\Omega |b,\Omega_{0}\right)I\left(\Omega \in M_{G}^{+}\right)$. Bayesian graphical lasso (Wang et al., 2012) assumes $\tilde{\pi }(\Omega )$ to be a product of independent exponential priors *Exp*(·|λ) and double-exponential priors *DE*(·|λ) on diagonal and off-diagonal elements of **Ω**, $\Omega ,\pi (\Omega |\lambda )\propto \prod_{j<k}DE\left(\omega_{jk}|\lambda \right)\prod_{j}Exp\left(\omega_{ii}|\lambda /2\right)I\left(\Omega \in M^{+}\right)$. Graphical spike-and-slab prior (Wang, 2015) replaces the double-exponential priors in Bayesian graphical lasso by spike-and-slab priors, $\pi \left(\Omega |G,v_{1},v_{0},\lambda \right)\propto \prod_{\{j,k\}\in E}N\left(\omega_{jk}|0,v_{1}\right)\prod_{\{j,k\}\notin E}N\left(\omega_{jk}|0,v_{0}\right)\prod_{j}Exp\left(\omega_{ii}|\lambda /2\right)I\left(\Omega \in M^{+}\right)$ where *v*_1_ ≫ *v*_0_. Priors on **Ω** can be defined either conditionally on the graph *G* or marginally; in what follows we do not use a model indicator parameter *G* but will infer the graph structures directly from the zero patterns in the precision matrices.

## 3. Gaussian graphical models with covariates

Let ***y***_1_, … , ***y***_*n*_ be *n* realizations of a random vector ***Y*** = (*Y*_1_, … , *Y*_*p*_). We assume an independent multivariate Gaussian distribution for each observation $y_{i}∼p\left(y_{i}|\Omega_{i}\right)=N\left(0,\Omega_{i}^{-1}\right)$ with the precision matrix $\Omega_{i}=\left[\omega_{ijk}\right]$, importantly, indexed by *i* = 1, … , *n*. A subject-level graph *G*_*i*_ = (*V*, *E*_*i*_) is embedded in the subject-level precision matrix $\Omega_{i}:\{j,k\}\in E_{i}$ if and only if $\omega_{ijk}\neq 0$.

Without further modeling assumptions, **Ω**_*i*_ cannot be estimated with a single observation *i*. Let ***x***_1_, … , ***x***_*n*_ be *n* realizations of covariates ***X*** = (1, *X*_1_, … , *X*_*q*_). Note that when ***X*** = 1 (i.e., there is no covaraites), the proposed GGMx is reduced to standard GGMs; more discussion of special cases of GGMx will be given later. We model **Ω***_i_* ≔ ***f***(***x***_*i*_) through a symmetric matrix-valued function ***f***(·), which is estimable as a population-level parameter shared across all observations.

### General construction of covariate-dependent priors.

The key is the construction of the function $f(\cdot )=\left[f_{jk}(\cdot )\right]$ such that $\Omega_{i}=f\left(x_{i}\right)$ is a PDM for any ***x***_*i*_, *i* = 1, … , *n*. Let ${ℳ}^{+}$ denote the collection of all such functions. This can be achieved by specifying a prior ***f*** ~ II that assigns positive mass only on functions that satisfy such requirement, $\Pi \left({ℳ}^{+}\right)=1$. We consider the following generalization of the prior density in (1) as,

(2) $$\pi (f)=\frac{\tilde{\pi }(f)I\left(f\in {ℳ}^{+}\right)}{\int \tilde{\pi }(f)I\left(f\in {ℳ}^{+}\right)df},$$

where $\tilde{\pi }$ is a distribution on matrix-valued functions. Note that the support of $\tilde{\pi }$ is not limited to ${ℳ}^{+}$, offering great flexibility in the choice of $\tilde{\pi }$. For example, we can start from independent distributions *a priori* such that $\tilde{\pi }(f)=\prod_{j\leq k}\tilde{\pi }\left(f_{jk}\right)$; using this construction, the marginal distribution $\tilde{\pi }\left(f_{jk}\right)$ need not be defined with a constrained range. Because of the deterministic relationship **Ω***_i_* = ***f*** (***x***_*i*_), prior π(***f***) induces a conditional prior on **Ω***_i_* given ***x**_i_*.

Two additional critical properties are desired for ***f***(·). (i) *Smoothness* — similar inputs should give rise to similar PDMs. Without smoothness, similar subjects may have vastly different networks, which is difficult to interpret in many applications including ours. (ii) *Sparsity* — π(***f***) should have positive probability on sparse PDMs. Sparsity is a common assumption in high-dimensional models including GGMs, which improves statistical efficiency and interpretability compared to dense models. In order to encourage sparsity of **Ω***i*, a positive mass has to be placed on sparse PDMs *a priori* because otherwise there will be zero mass on sparse PDMs *a posteriori* even if data strongly favor sparse PDMs. To equip ***f***(·) with these two properties, we decompose each off-diagonal element *f_jk_*(·) of ***f*** (·) into two components,

(3) $$f_{jk}\left(x_{i}\right)=g_{jk}\left(x_{i}\right)I\left(\left|g_{jk}\left(x_{i}\right)\right|>t_{jk}\right),\;\text{for}\;j<k,$$

where $g_{jk}(\cdot )$ is some smooth function, the hard thresholding $I\left(\left|g_{jk}\left(x_{i}\right)\right|>t_{jk}\right)$ promotes sparsity in $f_{jk}(\cdot )$, and *t_jk_* is a *random* threshold, which can be interpreted as a minimum effect size of *ω_ijk_*. Specifically, whenever $g_{jk}\left(x_{i}\right)$ is less than *t_jk_* in magnitude, the hard thresholding truncates $f_{jk}\left(x_{i}\right)$ to zero and hence induces a missing edge between nodes *j* and *k* for subject *i*. Our use of a thresholding function to induce sparsity on precision matrices **Ω***i*= ***f***(***x****_i_*) is novel and crucially different from conventional GGM priors including the G-Wishart prior and the graphical spike-and-slab prior (Wang, 2015): in order to construct observation-specific graphs, conventional priors would require a latent indicator for each potential edge and each observation, which would greatly increase the model complexity. For example, in our application with multiple myeloma dataset, conventional priors would need *n·p·*(*p*—1)/2 = 79,728 latent indicators whereas the proposed GGMx needs much fewer *p* · (*p* — 1)/2 = 528 thresholding parameters. Moreover, as will be introduced later, GGMx enables undirected graph interpolation for unseen covariates, a new feature that is difficult to obtain with conventional priors. Other choices of thresholding functions are possible such as soft thresholding and nonnegative garrote thresholding. The main motivation of choosing hard thresholding over the alternatives is its theoretical connection with mixture of non-local priors; see Section 4.

For the diagonal elements (inverse-partial-variance, Whittaker 2009) *f_jj_*(·) of ***f***(·), we assume the following model to ensure its nonnegativity,

(4) $$f_{jj}\left(x_{i}\right)=\exp\left\{g_{jj}\left(x_{i}\right)\right\}.$$

Note that unlike off-diagonal elements in (3), the diagonal element *f_jj_*(·) is not subject to thresholding.

**Remark 1** *Our formulation encompasses covariate-dependent priors on both the off-diagonal (inverse-covariance) and diagonal (inverse-partial-variance) elements, thus conducting both graphical and inverse-partial-variance regression, simultaneously.*

**Remark 2** *The proposed prior has two advantages over the more commonly used G-Wishart prior: (i) the induced prior on* **Ω***_i_ from* (2) *explicitly incorporates covariates* ***x***_*i*_ *and (ii) the normalizing constant of G-Wishart is not a constant with respect to graph G and therefore comparing two graphs requires explicit evaluation of the intractable normalizing constant whereas* *π*(***f***), *due to the thresholding function, does not have such complication.*

Given ***f***(·) and ***X***, the proposed GGMx satisfies *functional Markov properties*, e.g., the pairwise functional Markov property, which is stated formally in the following lemma.

**Lemma 1** *If f_jk_*(***X***) = 0, *then* $Y_{j}╨Y_{k}|Y_{rest}$, ***X** where* ***Y***_*rest*_ *is the subvector of* ***Y*** *without* *Y_j_* and *Y*_*k*_.

The proof of Lemma 1 directly follows from the fact that *f_jk_*(***X***) = 0 implies there is a missing edge between nodes *j* and *k* given covariates ***X***, which in turn implies that $Y_{j}╨Y_{k}|Y_{rest}$, ***X*** from standard GGM theory.

A natural choice of *g_jk_*(·) is a linear function $g_{jk}\left(x_{i}\right)=\beta_{jk}^{T}x_{i}$ although, in general, *g_jk_*(·) can be any smooth function. Given the limited sample size of the case study, we consider *g_jk_*(·) to be linear for parsimony (see Section 8 for a brief discussion on modeling a nonlinear *g*_*jk*_) and interpretability (***β**_jk_* are the rates of changes of *ω_ijk_* in ***x****_i_*). If the focus is on learning the graph structure and strength, i.e., the off-diagonal elements of **Ω***_i_*, one can further simplify the model by reducing diagonal elements *g_jj_*(***x***_*i*_) to be constant with respect to the covariates.

GGMx is a fairly flexible class of models and has at least five special cases (see Table 1). (i) If ***X*** only contains the intercept, then GGMx reduces to the case of the standard GGM because the graph is a function of a constant and hence is constant. (ii) If ***X*** is categorical, then GGMx is a multiple graphical model (also known as group-specific GGM) as the categorical covariate defines the groups. (iii) If ***X*** is univariate time points, then GGMx can be used for modeling time-varying GGMs (Zhou et al., 2010) by treating time as a covariate^1^. (iv) If the thresholds *t*_*jk*_’s are fixed to 0, then GGMx is a covariate-dependent GGM in which the strength of the graph varies continuously with the covariates but the structure is constant because a non-zero linear function is non-zero almost everywhere. (v) If ***X*** is a subset of ***Y***, then GGMx can be interpreted as a context-specific GGM (Nyman et al., 2017) where the graph structure varies with (discretized) ***X***.

**Priors.** We assign priors to ***β***_*jk*_ and *t*_*jk*_, which in turn define $\tilde{\pi }(f)$. We assume an independent multivariate Gaussian prior $\beta_{jk}∼\pi \left(\beta_{jk}\right)=N\left(\beta_{jk}|0,\tau_{jk}I_{q}\right)$. The thresholding parameter *t_jk_* can be interpreted as the minimum size of off-diagonal elements of **Ω***_i_*. Since its value is usually unknown in practice, we assign a truncated normal prior $t_{jk}∼\pi \left(t_{jk}\right)=N\left(\mu_{t},\sigma_{t}^{2}\right)I\left(t_{jk}>0\right)$ to reflect the uncertainty. As we will show in the next section, the priors of *β_jk_* and *t_jk_* induce a mixture of non-local priors on **Ω**_*i*_.

To complete the prior formulation, for the hyperparamter *τ_jk_*, we assign a hyperprior

$$\tau ={\left\{\tau_{jk}\right\}}_{j\leq k}∼\pi (\tau )=\frac{C_{\tau }\prod_{j\leq k}IG\left(\tau_{jk}|a_{\tau },b_{\tau }\right)}{\int C_{\tau }\prod_{j\leq k}IG\left(\tau_{jk}|a_{\tau },b_{\tau }\right)d\tau }$$

where *IG*(*a, b*) denotes an inverse-gamma density with shape *a* and scale *b*, and *C*_*τ*_ is the normalizing constant in (2),

$$C_{\tau }=\int \tilde{\pi }(f)I\left(f\in {ℳ}^{+}\right)df.$$

Including *C_τ_* in the prior of *π*(***τ***) serves to cancel out $C_{\tau }^{-1}$ in (2) so that the full conditional of *τ_jk_* is inverse-gamma. Similar cancellation trick has been used and thoroughly investigated in Bayesian graphical lasso (Wang et al., 2012).

**Figure 2:**
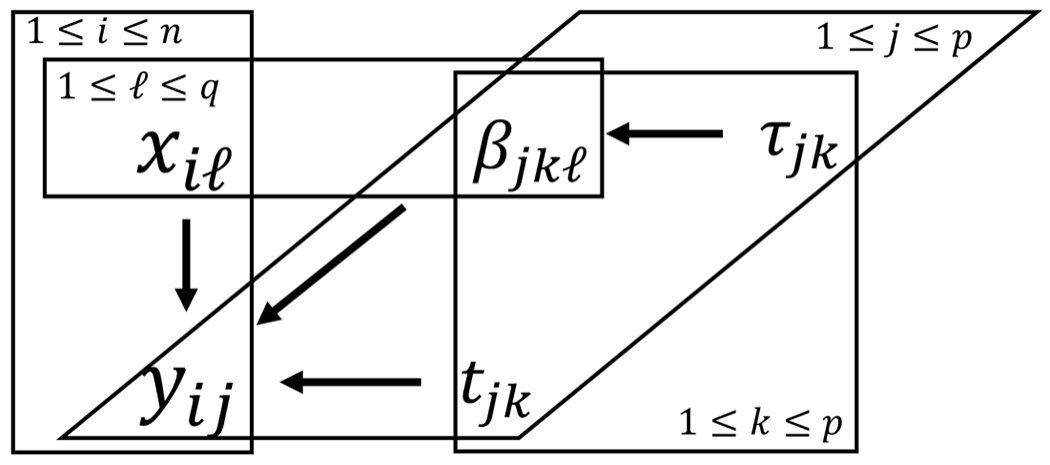
A schematic representation of GGMx.

A schematic representation of the proposed GGMx is provided in Figure 2.

## 4. Theoretical Properties

We establish a general result of the connection between the proposed prior of precision matrices induced by (2) and (3) and non-local alternative priors in GGM. A non-local prior assigns a vanishing density (under the alternative hypothesis) to the neighborhood of the null hypothesis. In variable selection contexts, this density vanishes around 0 and therefore shrinks small effect to zero, which is appealing because we are interested in a parsimonious estimation of the graph (i.e., a sparse network). Non-local priors have been shown, both theoretically and empirically, to have superior performance over local priors in various applications including hypothesis testing, high-dimensional sparse regression, and Bayesian networks (Johnson and Rossell, 2010, 2012; Altomare et al., 2013; Rossell and Telesca, 2017; Shin et al., 2018; Ni et al., 2019). However, to the best of our knowledge, all existing priors of sparse precision matrices in GGM (G-Wishart, Bayesian graphical lasso, and stochastic search structure learning prior) are local, i.e., *π*(**Ω**) does not approach 0 as *ω_jk_* → 0 for (*j, k*) ∈ *E*. Conceptually, local priors have a seemingly “contradictory” representation of one’s prior belief. On the one hand, (*j, k*) ∈ *E* suggests *ω_jk_* is non-zero. But on the other hand, local priors fail to assign zero mass at *ω_jk_* = 0; in fact, local priors often assign the maximum mass at zero. The practical implication of such “contradiction” is that local priors tend to favor denser models and be more susceptible to false discoveries compared to non-local priors especially for high-dimensional models like GGMx.

Let *π_θ_* and *π_t_* generically denote the priors for *θ_jk_* and $t_{jk},\theta_{jk}∼\pi_{\theta }\left(\theta_{jk}\right)$ and $t_{jk}∼\pi_{t}\left(t_{jk}\right)$. Let $T=\left[t_{jk}\right]$. We now show the connection between non-local priors and the proposed prior of the following general form,

$$\pi (\Omega |T)=\frac{\tilde{\pi }(\Omega |T)I\left(\Omega \in M^{+}\right)}{\int \tilde{\pi }(\Omega |T)I\left(\Omega \in M^{+}\right)d\Omega },$$

and

$$\tilde{\pi }(\Omega |T)=\overset{p}{\underset{j=1}{\prod }}\pi_{d}\left(\omega_{jj}\right)\underset{j<k}{\prod }\pi_{\omega |t}\left(\omega_{jk}|t_{jk}\right),$$

where

$$\omega_{jk}=\theta_{jk}I\left(\left|\theta_{jk}\right|>t_{jk}\right),\;\text{for}\;j<k.$$

Note that the equations above have no reference to covariates. We deliberately do so for clarity and generality; all the following theoretical results apply to the marginal distribution *π*(**Ω***_i_*) in GGMx by letting $\theta_{jk}=g_{jk}\left(x_{i}\right)=\beta_{jk}^{T}x_{i}$ and $\omega_{jj}=\exp\left\{g_{jj}\left(x_{i}\right)\right\}$. Conditional on *t_jk_*, the prior *π_θ_* induces a spike-and-slab mixture distribution,

$$\pi_{\omega |t}\left(\omega_{jk}|t_{jk}\right)=\rho \delta_{0}\left(\omega_{jk}\right)+(1-\rho ){\tilde{\pi }}_{\omega |t}\left(\omega_{jk}|t_{jk}\right)$$

where the mixture weight $\rho =\mathrm{Pr}\left(\left|\omega_{jk}\right|<t_{jk}|t_{jk}\right)$ is computed under the conditional distribution of *ω_jk_* induced by *π_θ_*(·) and hence is a function of *t_jk_* (not *ω_jk_*), and the slab is a truncated distribution,

$${\tilde{\pi }}_{\omega |t}\left(\omega_{jk}|t_{jk}\right)=\frac{\pi_{\theta }\left(\omega_{jk}\right)I\left(\left|\omega_{jk}\right|>t_{jk}\right)}{Pr\left(\left|\omega_{jk}\right|>t_{jk}|t_{jk}\right)}.$$

Slightly abusing the notations, let $\omega =\left(\omega_{1},\ldots ,\omega_{M}\right)=\left(\omega_{12},\ldots ,\omega_{1p},\omega_{23},\ldots ,\omega_{2p},\ldots ,\omega_{p-1,p}\right)$ be an *M*-dimensional vector containing upper-triangular elements of **Ω** with $M=\left(\begin{array}{c} p\\ 2 \end{array}\right)$. Let S⊆{1,…,M} denote the indices of non-zeros elements in **Ω** (or equivalently in ***ω***), i.e. *ω_m_* = 0 if and only if *m* ∈ *S^c^*. Then the conditional prior of **Ω** given ***T*** can be written as a mixture over all possible subsets *S*,

(5) $$\pi (\Omega |T)=\frac{1}{g(T)}I\left(\Omega \in M^{+}\right)\overset{p}{\underset{j=1}{\prod }}\pi_{d}\left(\omega_{jj}\right)$$

(6) $$\times \underset{S\in 2^{\left\{1,\ldots ,M\right\}}}{\sum }\underset{m\in S}{\prod }\pi_{\theta }\left(\omega_{m}\right)I\left(\left|\omega_{m}\right|>t_{m}\right)\underset{m\in S^{c}}{\prod }Pr\left(\left|\omega_{m}\right|<t_{m}|t_{m}\right)\delta_{0}\left(\omega_{m}\right),$$

where $g(T)=\int \tilde{\pi }(\Omega |T)I\left(\Omega \in M^{+}\right)d\Omega$ is the normalizing constant and 2^{1,…,*M*}^ is the power set of {1, … , *M*}. Our main theorem shows that under very mild conditions, the marginal prior *π*(**Ω**) is a discrete mixture of non-local priors. Before we present the main theorem, we first state a lemma that is useful in proving the theorem.

**Lemma 2** *E*[1/*g*(***T***)] < ∞ *if the distribution* *π_θ_*(·) of *θ*_*jk*_ *has positive mass around zero, i.e., there exists δ* > 0 *such that for any* *$0<\delta \prime <\delta ,\int_{-\delta \prime }^{\delta \prime }\pi_{\theta }(\theta )d\theta >0$,* *and the distribution* *π*_d_*(·)* *of ω_jj_ is not a point mass at zero, i.e.,* $\pi_{d}(\cdot )\neq \delta_{0}(\cdot )$*.*

**Proof** Consider

$$g(T)=\int \tilde{\pi }(\Omega |T)I\left(\Omega \in M^{+}\right)d\Omega =\mathrm{Pr}\left(\Omega \in M^{+}|T\right)\;>\mathrm{Pr}\left({\left\{\omega_{jj}>(p-1)\lambda \right\}}_{j=1}^{p},{\left\{\left|\omega_{jk}\right|\leq \lambda \right\}}_{j<k}|T\right),\;\forall \lambda \geq 0\;>\mathrm{Pr}\left({\left\{\omega_{jj}>(p-1)\lambda \right\}}_{j=1}^{p},{\left\{\left|\theta_{jk}\right|\leq \lambda \right\}}_{j<k}\right)\;=\overset{p}{\underset{j=1}{\prod }}Pr\left(\omega_{jj}>(p-1)\lambda \right)\underset{j<k}{\prod }Pr\left(\left|\theta_{jk}\right|\leq \lambda \right)\overset{\text{def}}{=}L(\lambda ).$$

The first inequality holds because diagonally dominant symmetric matrix is positive definite and the second inequality is true because $\left|\theta_{jk}\right|\leq \lambda$ implies $\left|\omega_{jk}\right|\leq \lambda$ by design and ***T*** is independent of *ω_jj_* and *θ_jk_*. If *π_θ_* has positive mass around zero and *π_d_* is not a point mass at zero, we can pick a sufficiently small (but positive) λ* > 0 such that the lower bound *L*(λ*) of *g*(***T***) is positive. Then it follows that *E*[1/*g*(***T***)] < ∞.

**Theorem 1** *The marginal prior* *π*(**Ω**) *is given by*

$$\pi (\Omega )=\underset{S\in 2^{\left\{1,\ldots ,M\right\}}}{\sum }\rho_{S}\pi_{S}(\Omega ),$$

*where π_S_*(**Ω**) *is the prior under the hypothesis* $H_{S}:\omega_{m}\neq 0,m\in S$ *and ω_m_ = 0, m ∈ S^c^. Moreover, π_S_*(**Ω**) *is a non-local prior for any* $S\in 2^{\left\{1,\ldots ,M\right\}}\backslash \emptyset$, *that is*, $\pi_{S}(\Omega )\to 0$ *as* $\omega_{m}\to 0$ *for m ∈ S, provided (i) Pr*(*t* = 0) = 0, (*ii*) *π_θ_*(·) *is bounded and has positive mass near 0, and* (*iii*) $\pi_{d}(\cdot )\neq \delta_{0}(\cdot )$.

**Proof** The marginal distribution of **Ω** is given by

$$\pi (\Omega )=\int \pi (\Omega |T)\pi_{t}(T)dT=I\left(\Omega \in M^{+}\right)\overset{p}{\underset{j=1}{\prod }}\pi_{d}\left(\omega_{jj}\right)\int \frac{1}{g(T)}\underset{j<k}{\prod }\pi_{\omega |t}\left(\omega_{jk}|t_{jk}\right)\pi_{t}(T)dT.$$

Let $m(\Omega )=I\left(\Omega \in M^{+}\right)\prod_{j=1}^{p}\pi_{d}\left(\omega_{jj}\right)$, then

$$\pi (\Omega )=\int m(\Omega )\frac{1}{g(T)}\underset{j<k}{\prod }\pi_{\omega |t}\left(\omega_{jk}|t_{jk}\right)\pi_{t}(T)dT\;=\int m(\Omega )\frac{1}{g(T)}\underset{j<k}{\prod }\left\{\mathrm{Pr}\left(\left|\omega_{jk}\right|<t_{jk}|t_{jk}\right)\delta_{0}\left(\omega_{jk}\right)+\pi_{\theta }\left(\omega_{jk}\right)I\left(\left|\omega_{jk}\right|>t_{jk}\right)\right\}\pi_{t}\left(t_{jk}\right)dT\;=\int m(\Omega )\frac{1}{g(T)}\underset{S\in 2^{\left\{1,\ldots ,M\right\}}\}}{\sum }\underset{m\in S}{\prod }\pi_{\theta }\left(\omega_{m}\right)I\left(\left|\omega_{m}\right|>t_{m}\right)\pi_{t}\left(t_{m}\right)\underset{m\in S^{c}}{\prod }\mathrm{Pr}\left(\left|\omega_{m}\right|<t_{m}|t_{m}\right)\delta_{0}\left(\omega_{m}\right)\pi_{t}\left(t_{m}\right)dT\;=\underset{S\in 2^{\left\{1,\ldots ,M\right\}}}{\sum }m(\Omega )E_{T}\left[\frac{1}{g(T)}\underset{m\in S}{\prod }I\left(\left|\omega_{m}\right|>t_{m}\right)\underset{m\in S^{c}}{\prod }\mathrm{Pr}\left(\left|\omega_{m}\right|<t_{m}|t_{m}\right)\right]\underset{m\in S}{\prod }\pi_{\theta }\left(\omega_{m}\right)\underset{m\in S^{c}}{\prod }\delta_{0}\left(\omega_{m}\right)\;\overset{\text{def}}{=}\underset{S\in 2\{1,\ldots ,M\}}{\sum }h_{S}(\Omega )=\underset{S\in 2\{1,\ldots ,M\}}{\sum }\int h_{S}(\Omega )d\Omega \times \frac{h_{S}(\Omega )}{\int h_{S}(\Omega )d\Omega }\overset{\text{def}}{=}\underset{S\in 2\{1,\ldots ,M\}}{\sum }\rho_{S}\times \pi_{S}(\Omega ).$$

We will show that for any *m* ∈ *S* and any sequence $\omega_{m}^{\left(n\right)}\to 0$ as $n\to \infty ,\pi_{S}\left(\Omega^{\left(n\right)}\right)\to 0$ as *n* → ∞ where **Ω**^(*n*)^ contains $\omega_{m}^{\left(n\right)}$ as an element. Note that

$$\frac{1}{g(T)}\underset{m\in S}{\prod }I\left(\left|\omega_{m}\right|>t_{m}\right)\underset{m\in S^{c}}{\prod }Pr\left(\left|\omega_{m}\right|<t_{m}|t_{m}\right)\leq \frac{1}{g(T)}.$$

Since *E*[1/*g*(***T***)] < ∞ due to conditions (ii) - (iii) and Lemma 2, and $\lim_{n\to \infty }I(\left|\omega_{m}^{\left(n\right)}\right|>t_{m})=0$ almost surely due to condition (i), then by dominated convergence theorem, we have

$$\underset{n\to \infty }{\lim}E_{T}\;\left[\frac{1}{g(T)}I\left(\left|\omega_{m}^{\left(n\right)}\right|>t_{m}\right)\underset{m\prime \in S,m\prime \neq m}{\prod }I\left(\left|\omega_{m\prime }\right|>t_{m\prime }\right)\underset{m\in S^{c}}{\prod }Pr\left(\left|\omega_{m}\right|<t_{m}|t_{m}\right)\right]\;=E_{T}\;\left[\frac{1}{g(T)}\left\{\underset{n\to \infty }{\lim}I\left(\left|\omega_{m}^{\left(n\right)}\right|>t_{m}\right)\right\}\underset{m\prime \in S,m\prime \neq m}{\prod }I\left(\left|\omega_{m\prime }\right|>t_{m\prime }\right)\underset{m\in S^{c}}{\prod }Pr\left(\left|\omega_{m}\right|<t_{m}|t_{m}\right)\right]\;=0$$

Finally, condition (ii) renders $\pi_{S}\left(\Omega^{\left(n\right)}\right)\to 0$.

Conditions (i) - (iii) in Theorem 1 are very mild and satisfied by a wide range of *π_t_*, *π_θ_*, and *π_d_*. Condition (i) is trivially satisfied if *π_t_* is continuous (e.g., gamma, inverse-gamma, log-normal, and truncated normal distributions). Condition (ii) holds for Cauchy, normal, and most of the scale mixtures of normal distributions such as Laplace, normal-gamma, and t distributions. Condition (iii) only excludes point mass at zero *δ*_0_(·) from all the possible choices of *π_d_*(·).

### A simple illustrative example

**Figure 3:**
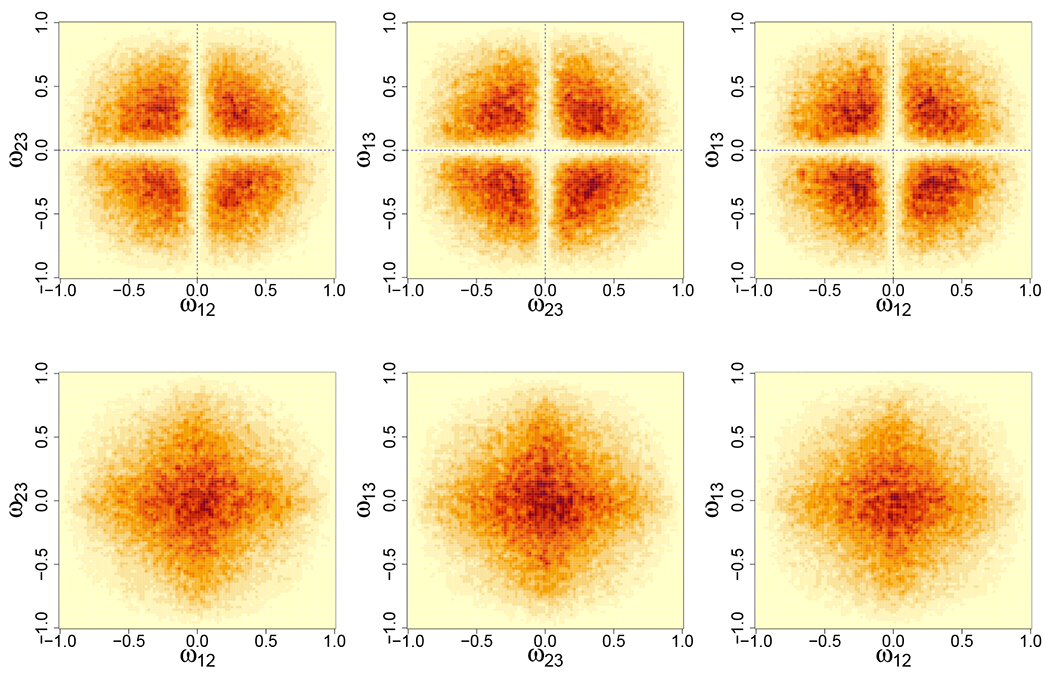
Non-local (top) and local (bottom) prior distributions of **Ω**.

As a concrete example, *π_S_*(**Ω**) is non-local under the prior distributions specified in Section 3, namely, $\pi_{\theta }\left(\theta_{jk}\right)=N(0,\tau ),\pi_{d}\left(\omega_{jj}\right)=\text{log-normal}(0,\tau )$, and $\pi_{t}\left(t_{jk}\right)=N\left(\mu_{t},\sigma_{t}^{2}\right)I\left(t_{jk}>0\right)$. To visualize the proposed non-local prior, we consider a small precision matrix with *p* = 3 and perform a prior simulation to generate **Ω** from *π*(**Ω**), the procedure of which is a special case of the posterior simulation procedure (ignoring the likelihood) to be described in Section 5. We visualize *π_S_*(**Ω**) for *S* = {1, … , *M*}, i.e., a complete graph. The marginal densities of pairs of off-diagonal elements of **Ω** (normalized to partial correlations) are depicted in the top panel of Figure 3 which show vanishing density as *ω_jk_* approaches 0. By contrast, a local prior on **Ω** (simulated by fixing *t_jk_* = 0) has an increasing density as *ω_jk_* approaches 0 as shown in the bottom panel of Figure 3.

**Remark** The connection between non-local priors and random thresholding has been investigated in the regression context (Rossell and Telesca, 2017; Ni et al., 2019). We make a nontrivial extension to precision matrix estimation for undirected GGMs. One major difference between our theory and those in Rossell and Telesca (2017) and Ni et al. (2019) is the complexity of the intractable prior normalizing constant *g*(***T***) in (5). Intractable prior normalizing constant is a common challenge in standard Bayesian GGMs (Dobra et al., 2011; Wang et al., 2012; Wang, 2015), both theoretically and computationally. In order to show the equivalence between non-local priors and random thresholding for GGMs, we make extra assumptions, i.e., *π_θ_*(·) has positive mass around zero and $\pi_{d}(\cdot )\neq \delta_{0}(\cdot )$, in order to bound *E*[1/*g*(***T***)]. These mild assumptions are not required in previous works. Also note that Rossell and Telesca (2017) truncates probability density whereas we threshold the random variables. Consequently, the resulting marginal prior of Rossell and Telesca (2017) is a non-local prior while ours is a discrete mixture of the non-local prior and point mass at 0. Computationally, the issue of intractable normalizing constant is resolved by a carefully designed MCMC algorithm, which will be discussed in the next section.

## 5. Posterior Inference

The proposed GGMx is parameterized by three sets of parameters ${\left\{\beta_{jk}\right\}}_{j\leq k},{\left\{t_{jk}\right\}}_{j<k}$, and ${\left\{\tau_{jk}\right\}}_{j\leq k}$. The joint posterior distribution of these parameters is given by,

$$p\left({\left\{\beta_{jk}\right\}}_{j\leq k},{\left\{t_{jk}\right\}}_{j<k},{\left\{\tau_{jk}\right\}}_{j\leq k}|{\left\{y_{i},x_{i}\right\}}_{i=1}^{n}\right)\;\propto \overset{n}{\underset{i=1}{\prod }}N\left(y_{i}|0,\Omega_{i}\right)\underset{j<k}{\prod }N\left(t_{jk}|\mu_{t},\sigma_{t}^{2}\right)I\left(t_{jk}>0\right)\underset{j\leq k}{\prod }N\left(\beta_{jk}|0,\tau_{jk}I_{q}\right)IG\left(\tau_{jk}|a_{\tau },b_{\tau }\right),$$

where the right-hand side of this equation depends on ***x**_i_* through **Ω**_*i*_ = ***f*** (***x***_*i*_) and ***f***(·) is defined by ${\left\{\beta_{jk}\right\}}_{j\leq k}$ and ${\left\{t_{jk}\right\}}_{j<k}$. The posterior inference of the model parameters is carried out by MCMC. We need to carefully choose a proposal distribution that can propose $f\in {ℳ}^{+}$ efficiently. This is not a trivial task because the probability that we generate $f\in {ℳ}^{+}$ is practically zero if we propose ***β**_jk_* and *t_jk_* from naive proposals such as standard random walks. Here, we introduce a proposal that always proposes $f\in {ℳ}^{+}$.

For illustration, suppose we are currently updating the (*j, k*)th element of **Ω**_*i*_. Let ***ω***_*i,−k,k*_ denote the *k*th column of **Ω**_*i*_ without the *k*th row and let **Ω**_*i,−k,−k*_ denote the submatrix of **Ω**_*i*_ without the *k*th row and column. Let $\phi_{ik}=\omega_{ikk}-u_{ik}$ with $u_{ik}=\omega_{i,-k,k}^{T}\Omega_{i,-k,-k}^{-1}\omega_{i,-k,k}$. We first propose new $\beta_{jk\ell }^{*}$ and $t_{jk}^{*}$ from some proposal densities $q_{\beta }\left(\beta_{jk\ell }^{*}|\beta_{jk\ell }\right)$ and $q_{t}\left(t_{jk}^{*}|t_{jk}\right)$ such as random walks for ℓ = 1, … , *q*+1. The resulting new values of *ω_i,−k,k_* and *u_ik_* are denoted by $\omega_{i,-k,k}^{*}$ and $u_{ik}^{*}$. Notice that **Ω**_*i*_ is positive definite if and only if *ϕ_ik_* > 0 for *k* = 1, … , *p*. This is due to the Sylvester’s criterion that a symmetric matrix is positive definite if and only if all of the leading principal minors are positive. Without loss of generality, assuming *k* is the last column and all previous principal minors are positive, and assuming covariates *x_i_* are positive. Then the last leading principal minor $\det\left(\Omega_{i}\right)=\left(\omega_{ikk}-u_{ik}\right)\;\det\left(\Omega_{i,-k,-k}\right)$ is positive if and only if $\omega_{ikk}-u_{ik}>0$. Therefore, in order to ensure positive definiteness of **Ω**_*i*_, ∀*i* when updating its (*j, k*)th element, we will additionally propose a new $\beta_{kk\ell }^{*}$ such that $\omega_{ikk}^{*}=\exp\left\{g_{kk}^{*}\left(x_{i}\right)\right\}>u_{ik^{*}}$ where $g_{kk}^{*}\left(x_{i}\right)=x_{i\ell }\beta_{kk\ell }^{*}+\sum_{\ell \prime \neq \ell }x_{i\ell \prime }\beta_{kk\ell \prime }$. The solution to this inequality for all *i* is the constraint that the proposal of $\beta_{kk\ell }^{*}$ needs to respect. Specifically, we will propose $\beta_{kk\ell }^{*}∼q_{\beta }\left(\beta_{kk\ell }^{*}|\beta_{kk\ell }\right)I\left(\beta_{kk\ell }^{*}\in S_{k\ell }^{*}\right)$ where

$$S_{k\ell }^{*}=\left\{\beta |\beta >\underset{i}{\max}\;\left(\frac{\log\left(u_{ik}^{*}\right)-\sum_{\ell \prime \neq \ell }x_{i\ell \prime }\beta_{kk\ell \prime }}{x_{i\ell }}\right)\right\}.$$

We summarize the property of the proposal density in the following proposition, of which the proof is given by the proceeding paragraph.

**Proposition 1** *The proposal density* $q\left(\beta_{jk\ell }^{*},t_{jk}^{*},\beta_{kk\ell }^{*}|\beta_{jk\ell },t_{jk},\beta_{kk\ell }\right)=q_{\beta }\left(\beta_{jk\ell }^{*}|\beta_{jk\ell }\right)q_{t}\left(t_{jk}^{*}|t_{jk}\right)q_{\beta }\left(\beta_{kk\ell }^{*}|\beta_{kk\ell }\right)I\left(\beta_{kk\ell }^{*}\in S_{k\ell }^{*}\right)$ *and the full conditional density* $p\left(\beta_{jk\ell }^{*},t_{jk}^{*},\beta_{kk\ell }^{*}|\cdot \right)$ *have the same support*.

We now provide the MCMC (Metropolis-within-Gibbs) algorithm below; its validity is guaranteed by Proposition 1 and standard MCMC theory.

### The MCMC Algorithm.

Initialize model parameters. Repeat the following steps until practical convergence.

(I) Update precision matrices **Ω**_*i*_. Scanning through each column *k* = 1, … , *p*, each row *j* ≠ *k*, and each covariate *ℓ* = 1, … , *q* + 1, we propose $\beta_{jk\ell }^{*},t_{jk}^{*}$, and $\beta_{kk\ell }^{*}$ from $q_{\beta }\left(\beta_{jk\ell }^{*}|\beta_{jk\ell }\right),q_{t}\left(\log\;t_{jk}^{*}|\log\;t_{jk}\right)$, and $q_{\beta }\left(\beta_{kk\ell }^{*}|\beta_{kk\ell }\right)I\left(\beta_{kk\ell }^{*}\in S_{k\ell }^{*}\right)$ where $q_{t}\left(\log\;t_{jk}^{*}|\log\;t_{jk}\right)=N\left(\log\;t_{jk}^{*}|\log\;t_{jk},\eta_{t}^{2}\right),q_{\beta }\left(\beta_{jk\ell }^{*}|\beta_{jk\ell }\right)=N\left(\beta_{jk\ell }^{*}|\beta_{jk\ell },\eta_{\beta }^{2}\right)$ and $q_{\beta }\left(\beta_{kk\ell }^{*}|\beta_{kk\ell }\right)=N\left(\beta_{kk\ell }^{*}|\beta_{kk\ell },\eta_{\beta }^{2}\right)$. We accept the proposal with probability min(1, *α*) where

$$\alpha =\frac{\prod_{i=1}^{n}p\left(y_{i}|\Omega_{i}^{*}\right)\pi \left(\beta_{jk\ell }^{*}\right)\pi \left(t_{jk}^{*}\right)\pi \left(\beta_{kk\ell }^{*}\right)q_{\beta }\left(\beta_{jk\ell }|\beta_{jk\ell }^{*}\right)q_{t}\left(t_{jk}|t_{jk}^{*}\right)q_{\beta }\left(\beta_{jk\ell }|\beta_{jk\ell }^{*}\right)I\left(\beta_{kk\ell }\in S_{k\ell }\right)}{\prod_{i=1}^{n}p\left(y_{i}|\Omega_{i}\right)\pi \left(\beta_{jk\ell }\right)\pi \left(t_{jk}\right)\pi \left(\beta_{kk\ell }\right)q_{\beta }\left(\beta_{jk\ell }^{*}|\beta_{jk\ell }\right)q_{t}\left(t_{jk}^{*}|t_{jk}\right)q_{\beta }\left(\beta_{kk\ell }^{*}|\beta_{kk\ell }\right)I\left(\beta_{kk\ell }^{*}\in S_{k\ell }^{*}\right)}.$$

The proposal standard deviations $\eta_{t}^{2}$ and $\eta_{\beta }^{2}$ can be set to achieve desired acceptance rate (say, 20%-40%).

(II) Update the hypervariances *τ_jk_* from the inverse-gamma full conditional, $\tau_{jk}∼IG\left(a_{\tau }+1/2,b_{\tau }+\beta_{jk\ell }^{2}/2\right)$.

### Graph estimation.

A point estimate of *G_i_* can be obtained by thresholding the posterior probability of inclusion. Specifically, we select $\{j,k\}\in E_{i}$ if $Pr\left(\{j,k\}\in E_{i}|y_{i},x_{i}\right)>c$ where *c* ∈ [0, 1] is the probability cutoff^2^ . The posterior probability of inclusion can be approximated by the MCMC samples,

$$Pr\left(\{j,k\}\in E_{i}|y_{i},x_{i}\right)=Pr\left(\omega_{ijk}\neq 0|y_{i},x_{i}\right)\approx \frac{1}{R}\overset{R}{\underset{r=1}{\sum }}I\left\{\omega_{ijk}^{\left(r\right)}\neq 0\right\},$$

where the superscript (*r*) indexes the posterior samples.

### Graph interpolation.

Since the precision matrix **Ω**_*i*_ = ***f*** (***x***_*i*_) is modeled as a function of ***x**_i_*, we can interpolate a graph G^*^ = (V, E^*^) for an unseen observation at covariates x^*^. It is achieved through the posterior predictive distribution of ***f***(·), which can be approximated by the MCMC samples,

$$Pr\left(\{j,k\}\in E^{*}|y,x,x^{*}\right)=Pr\left\{f_{jk}\left(x^{*}\right)\neq 0|y,x,x^{*}\right\}\approx \frac{1}{R}\overset{R}{\underset{r=1}{\sum }}I\left\{f_{jk}^{\left(r\right)}\left(x^{*}\right)\neq 0\right\}.$$

Graph interpolation requires covariates x^*^ only, since the right-hand side of the equation above does not depend on y^*^. In practice, this is a desirable property. For example, one can predict the gene network for new patients without sequencing the whole genome; the measurement of covariates (e.g., blood biomarkers) will suffice.

## 6. Simulations

### 6.1 Simulation Setup

We assessed the utility and operating characteristics of GGMx in seven simulation scenarios with different levels of sparsity and types of covariates. The same size of the dataset in application was used: *n* = 151, *p* = 33, and *q* = 2 (*q* was set to 1 for the last scenario). Note that even with a moderate dataset, the number of parameters $\left(\beta_{jk},t_{jk},\tau_{jk}\right)$ that need to be estimated is $\frac{p(p+1)(q+2)}{2}+\frac{p(p-1)}{2}=2,772$, which is substantially larger than the sample size. We focused on graph structure learning in the first five scenarios by assuming constant diagonal elements *g_jj_*(·) for simplicity; non-constant case (i.e., simultaneous inverse-partial-variance and graphical regression) will be considered in the last two scenarios. We fixed the probability cutoff *c* to be 0.5 in all scenarios.

#### Scenario I.

We generated the simulated data from our model. We randomly set 2% of *β_jkℓ_* for *j* < *k* to be ±1 with equal probability. We set *t_jk_* = 0.5 and all the diagonal elements of **Ω**_*i*_ to be 1. The covariate *x_ij_* was generated from an uniform distribution $x_{ij}\overset{iid}{∼}U(-1,1)$. The resulting precision matrix **Ω**_*i*_ might not be positive definite for all observations *i* = 1, … , *n*. We repeated the process until **Ω**_*i*_ > 0, ∀*i*. Then the observation ***y**_i_* was drawn from normal $y_{i}\overset{ind}{∼}N\left(0,\Omega_{i}^{-1}\right)$. Using the same procedure, we generated a similar independent dataset with sample size 50 for testing graph interpolation of GGMx.

#### Scenario II.

**Figure 4:**
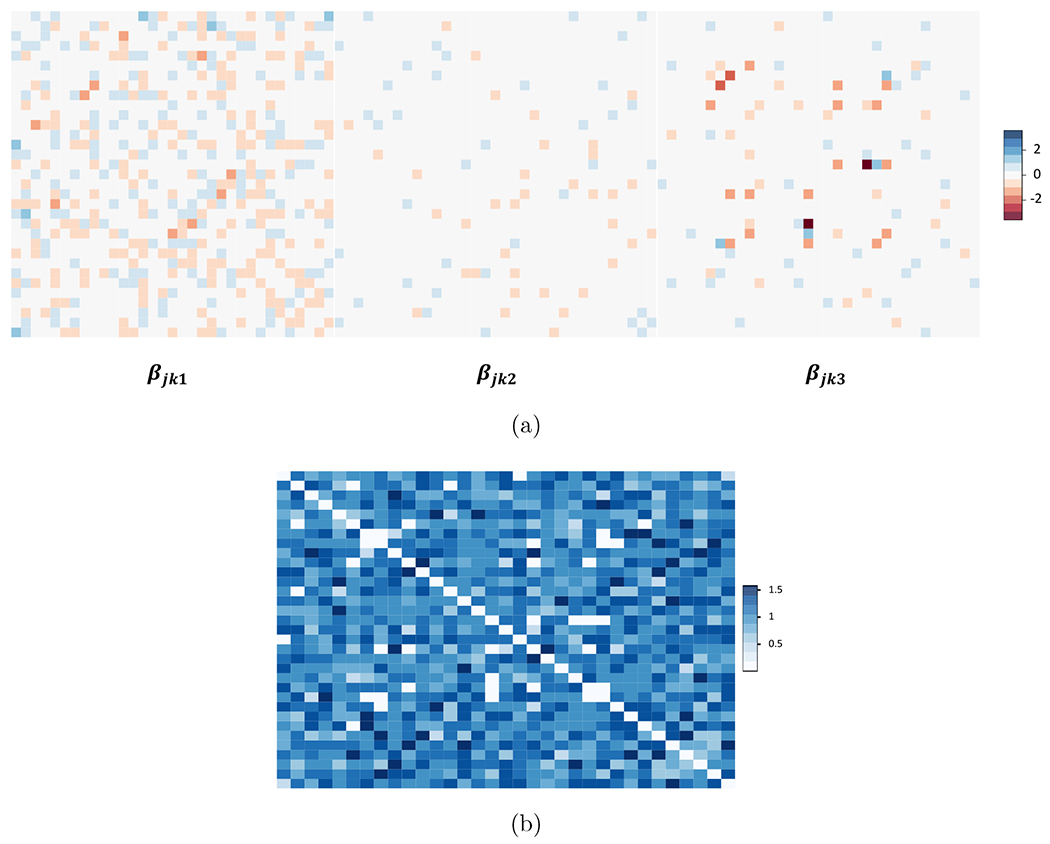
Simulation truths for Scenario II. Heatmaps of (a) true *β_jkl_*’s and (b) true *t*_*jk*_. They are one posterior draw from GGMx applied to the multiple myeloma data.

The procedure in Scenario I was inefficient to generate a denser network. In addition, it may not mimic well the data in application. In this scenario, we used one posterior draw from GGMx applied to the multiple myeloma data as simulation truth. The true *β_jkl_*’s are shown as heatmaps in Figure 4a where *ℓ* = 1 corresponds to the intercept and *ℓ* = 2, 3 correspond to the two covariates. Since the heatmap of *β*_*jk*1_ is denser than those of *β*_*jk*2_ and *β*_*jk*3_, there were more nearly constant edges than highly varying edges. The true *t_jk_*’s are shown in Figure 4b. The covariates ***x**_i_* of the multiple myeloma dataset was used. And ***y**_i_* was drawn from the model $y_{i}\overset{ind}{∼}N\left(0,\Omega_{i}^{-1}\right)$ with **Ω**_*i*_ = ***f***(***x**_i_*).

#### Scenario III.

This scenarios considered a simulation truth from an ordinary GGM, i.e. **Ω**_*i*_ = **Ω**, ∀*i*. We generated a true **Ω** as follows.

1. Generate an Erdös-Rényi graph *G* with connecting probability 5%.
2. Set the diagonal entries of **Ω** to 1. For each edge {*j, k*} in *G*, draw corresponding off-diagonal entrie *ω_jk_* uniformly in [−1, −0.5] ∪ [0.5, 1].
3. Since **Ω** might not be positive definite, we kept adding 0.1***I*** to **Ω** until **Ω** became positive definite. The resulting partial correlations were less than 0.4 in magnitude. Then we simulated $y_{i}\overset{iid}{∼}N\left(0,\Omega^{-1}\right)$ and $x_{ij}\overset{iid}{∼}U(-1,1)$. GGMx took the independently generated *x_ij_* as covariates, which were pure “noises” for constructing the graph of ***y**_i_*.

#### Scenario IV.

We extended Scenario III to multiple graphs with *C* = 3 groups. The sample size of each group was *n*_1_ = 50, *n*_2_ = 50, and *n*_3_ = 51. Graph *G*_1_ was generated as an Erdös-Rényi graph with connecting probability 10%, which led to 63 edges. We randomly turned 3 edges on and 3 edges off from *G*_1_ to obtain *G*_2_ and similarly constructed *G*_3_ from *G*_2_. As a result, each pair of (*G*_1_, *G*_2_) and (*G*_2_, *G*_3_) shares about 90% edges whereas (*G*_1_, *G*_3_) shares about 80% edges. Then given graphs, the precision matrices and observations ***y**_i_* were generated in the same way as Scenario III. To apply GGMx in this setting, we let *x_ij_* be a binary indicator such that *x_ij_* = 1 if observation *i* belongs to group *j* for *j* = 1, 2 and *x_ij_* = 0 for *j* = 1, 2 if observation *i* belongs to group 3.

#### Scenario V.

We have considered continuous covariates (Scenarios I-II), a discrete covariate (Scenarios IV), or no relevant covariates (Scenario III). Here, we included a scenario with one continuous covariate and one discrete covariate. We generated the data by following Scenario I with one covariate replaced by a *Bernoulli*(0.5) variable and the corresponding coefficients *β_jkℓ_*’s set to ±0.5 with equal probability.

#### Scenario VI.

We considered a scenario without assuming *ω_ijj_* to be a constant; instead we set *g_jj_*(***x***_*i*_) = 0.1 + 0.2*x*_*i*1_ + 0.2*x*_*i*2_ and $\omega_{ijj}=\exp\left\{g_{jj}\left(x_{i}\right)\right\}$. For off-diagonal elements, we randomly included 2% of the edges and the corresponding *β_jkl_* for *j* < *k* was set to be 0.7. The covariate *x_iℓ_* was generated from $x_{i\ell }\overset{iid}{∼}2Beta(2,1)$. The resulting precision matrix **Ω**_*i*_ might not be positive definite for all observations *i* = 1, … , *n*. We repeated the process until **Ω**_*i*_ > 0, ∀*i*. Then the observation ***y**_i_* was drawn from normal $y_{i}\overset{ind}{∼}N\left(0,\Omega_{i}^{-1}\right)$.

#### Scenario VII.

To illustrate GGMx can be used to recover time-varying GGM, we reduced the number of covariate to *q* = 1 from Scenario VI.

### 6.2 Methods under Consideration

We compared the proposed GGMx with six competing methods: Bayesian Gaussian graphical models (Mohammadi et al., 2015), graphical lasso (Friedman et al., 2008), kernel graphical lasso (Liu et al., 2010a), fused graphical lasso, group graphical lasso (Danaher et al., 2014), and Bayesian multiple Gaussian graphical model (Shaddox et al., 2018).

Bayesian Gaussian graphical models (BGGMs) assume i.i.d. multivariate Gaussian likelihood and the G-Wishart prior on the precision **Ω** ~ *W_G_*(*b, **D***) and a uniform prior on the graph *G*. G-Wishart prior is conjugate to the multivariate Gaussian likelihood. However, due to intractable prior normalizing constant of G-Wishart prior, non-trivial MCMC algorithm is required for posterior inference. We use an efficient trans-dimensional MCMC algorithm proposed by Mohammadi et al. (2015) based on a continuous-time birth-death process.

Graphical lasso (glasso) is a penalized likelihood approach that maximizes the objective function $\log\;|\Omega |-\mathrm{tr}(S\Omega )-\lambda ∥\Omega {∥}_{1}$ where ***S*** is the sample covariance matrix. The first two terms are the Gaussian log-likelihood and the last term is an *ℓ*_1_ penalty, which induces sparsity in **Ω**. The optimization is solved using a coordinate descent algorithm.

Both BGGM and glasso assume i.i.d. sampling and are designed to infer networks that do not change with covariates. For a more fair comparison, we implemented the kernel graphical lasso (k-glasso) approach outlined in Liu et al. (2010a). K-glasso is a modification of glasso with the sample covariance matrix ***S*** replaced by a covariate-dependent covariance matrix via kernel smoothing. Specifically, let

$$S(x)=\overset{n}{\underset{i=1}{\sum }}K\;\left(\frac{\left\|x-x_{i}\right\|}{h}\right)\left(y_{i}-\mu (x)\right){\left(y_{i}-\mu (x)\right)}^{T}/\overset{n}{\underset{i=1}{\sum }}K\;\left(\frac{\left\|x-x_{i}\right\|}{h}\right),$$

with

$$\mu (x)=\overset{n}{\underset{i=1}{\sum }}K\;\left(\frac{\left\|x-x_{i}\right\|}{h}\right)\;y_{i}/\overset{n}{\underset{i=1}{\sum }}K\;\left(\frac{\left\|x-x_{i}\right\|}{h}\right),$$

where || · || is the Euclidean norm, *h* > 0 is the bandwidth, and *K*(·) is a Gaussian kernel. Then a sparse estimate of **Ω**_*i*_ is obtained by applying glasso with $S=S\left(x_{i}\right),{\hat{\Omega }}_{i}={\mathrm{arg min}}_{\Omega }\left\{\log|\Omega |-\mathrm{tr}\left(S\left(x_{i}\right)\Omega \right)-\lambda_{i}∥\Omega {∥}_{1}\right\}$.

As pointed out in Section 3, the proposed GGMx is a multiple graphical model when the covariates are categorical. Multiple graphical models assume that observations are divided into *C* groups. The goal is to jointly estimate group-specific sparse precision matrices **Ω**^(*c*)^, *c* = 1, … , *C*. Since the grouping of observations can be represented by a categorical variable, GGMx is able to learn group-specific graphs. For comparison, we consider three alternative multiple graphical model approaches, the two penalized approaches proposed in Danaher et al. (2014), fused graphical lasso (FGL) and group graphical lasso (GGL), and the Bayesian multiple Gaussian graphical model (MGGM) proposed by Shaddox et al. 2018. Both penalized algorithms maximize the following objective with respect to positive definite matrices ${\left\{\Omega^{\left(c\right)}\right\}}_{c=1}^{C}$,

$$\overset{C}{\underset{c=1}{\sum }}n_{c}\left\{\log\;\left|\Omega^{\left(c\right)}\right|-\mathrm{tr}\left(S^{\left(c\right)}\Omega^{\left(c\right)}\right)\right\}-P\left({\left\{\Omega^{\left(c\right)}\right\}}_{c=1}^{C}\right),$$

where *n_c_* is the sample size of group *c*, ***S***^(*c*)^ is the sample covariance matrix of group *c*, and *P*(·) is a penalty that encourages sparsity and similarity of ${\left\{\Omega^{\left(c\right)}\right\}}_{c=1}^{C}$. The penalty is chosen to be $\lambda_{1}\sum_{c=1}^{C}\sum_{j\neq k}\left|\omega_{jk}^{\left(c\right)}\right|+\lambda_{2}\sum_{c<c\prime }\sum_{j,k}\left|\omega_{jk}^{\left(c\right)}-\omega_{jk}^{\left(c\prime \right)}\right|$ for FGL and $\lambda_{1}\sum_{c=1}^{C}\sum_{j\neq k}\left|\omega_{jk}^{\left(c\right)}\right|+\lambda_{2}\sum_{j\neq k}\sqrt{\sum_{c=1}^{C}\omega_{jk}^{(c)2}}$ for GGL.

Finally, MGGM uses local priors on sparse precision matrices (Wang, 2015) and can be thought as the local prior counterpart of the proposed method for the multiple graphs setting; comparisons with this method only pertain to Scenario IV.

For GGMx, we set the hyperparameters, *a_τ_* = *b_τ_* = 10^−1^, *μ_t_* = 1, and *σ_t_* = 0.2; these choices will be tested in sensitivity analyses at the end of this section. Both GGMx and BGGM were run for 10,000 iterations with 5,000 burn-in. The regularization parameter of glasso was selected by the stability approach (Liu et al., 2010b) implemented in the R package huge. The tuning parameters λ_1_ and λ_2_ of FGL and GGL were selected based on the approximated Akaike Information Criterion (AIC) as suggested by Danaher et al. (2014). A 20 × 20 grid evenly spaced between 0.05 and 0.5 for λ_1_, and between 0.001 and 0.01 for λ_2_, was used. Likewise, the tuning parameters λ_*i*_ and *h*_*i*_ of k-glasso were also selected based on AIC on a 20 × 20 grid [0.1, 1] × [0.1, 1] for each observation *i* = 1, … , *n*. All results were based on 50 repeat simulations.

### 6.3 Simulation Results

**Figure 5:**
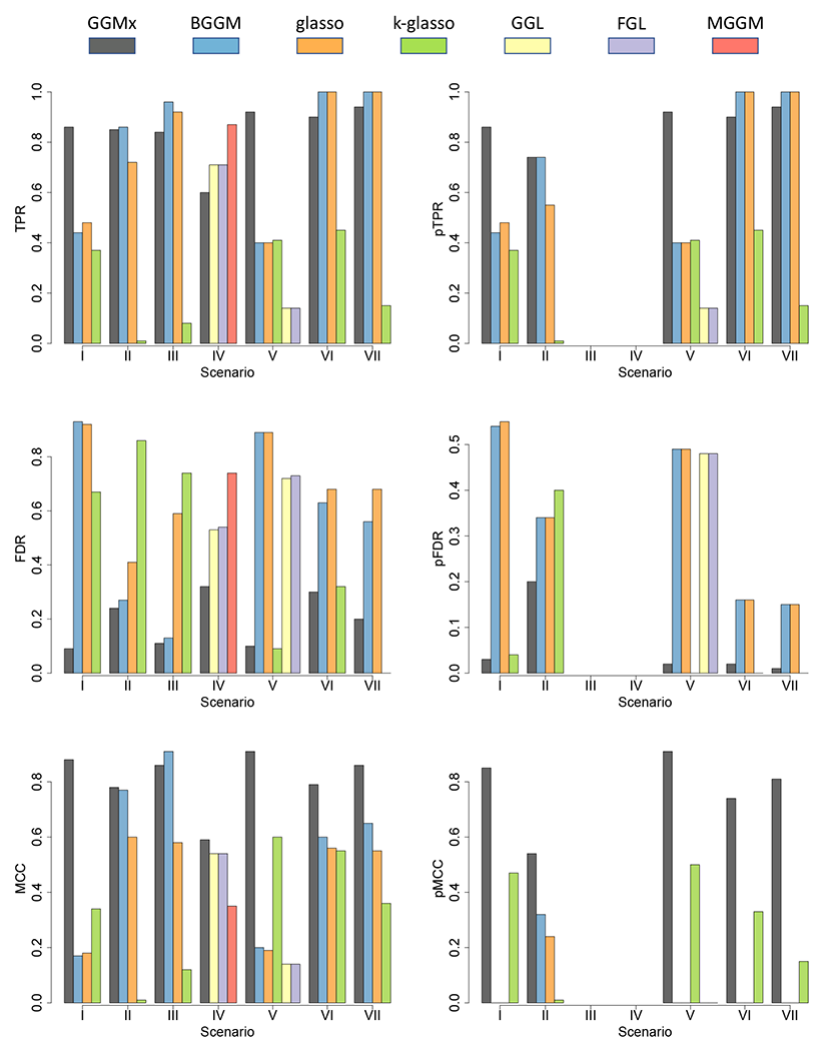
Simulations. Operating characteristics averaged over 50 repeat simulations under seven scenarios. Graph interpolation is not shown as it is similar to graph estimation. pMCC is 0 when no missing edge is detected. pTPR, pFDR, and pMCC are not available for Scenarios III and IV.

To assess the graph recovery performance, we computed true positive rate (TPR), false discovery rate (FDR), and Matthews correlation coefficient (MCC),

$$\text{TPR}=\frac{\text{TP}}{\text{TP}+\text{FN}},\;\text{FDR}=\frac{\text{FP}}{\text{TP}+\text{FP}},\;\text{MCC}=\frac{\text{TP}\times \text{TN}-\text{FP}\times \text{FN}}{\sqrt{\left(\text{TP}+\text{FP})(\text{TP}+\text{FN})(\text{TN}+\text{FP})(\text{TN}+\text{FN}\right)}},$$

where TP, FP, TN, and FN stand for true positives, false positives, true negatives, and false negatives. MCC takes value between −1 and 1 with 1 being perfect graph recovery and 0 being random guess. In addition, we scrutinized the edges with inclusion probability that is considerably affected by the covariates’ value. Hence, we introduced another three measures: partial TPR (pTPR), partial FDR (pFDR), and partial MCC (pMCC) which are simply TPR, FDR, and MCC restricted to the edges with true frequency of inclusion across observations between 0.1 and 0.9. We report all the metrics in Figure 5. Overall, GGMx had robust, superior performance (with high true positive and low false discovery rates) across all scenarios.

In Scenario I, GGMx clearly outperformed BGGM and glasso in all six measures. This was expected because the data were generated from the proposed model and all edges were associated with covariates. BGGM and glasso assume i.i.d. sampling and therefore did not perform well. Although k-glasso was much better than BGGM and glasso, it is clear that GGMx performed significantly better than k-glasso in all metrics. In addition, GGMx can interpolate graph structure given new covariates. The results of graph interpolation (not shown) were very similar to those of graph estimation.

In Scenario II, it appeared that BGGM was comparable to GGMx in TPR, FDR and MCC. This is because in the simulation truth, there were much more nearly constant edges than highly varying edges. In many cases including the application, it is interesting to focus on highly varying edges as they are most differential across observations. Not surprisingly, GGMx had favorable performance compared to BGGM and glasso in terms of pTPR, pFDR, and pMCC. K-glasso was not able to pick up the signals in this scenario, which mimicked the real data.

In Scenario III where there was no relationship between graph and covariates, BGGM outperformed GGMx, glasso, and k-glasso. But GGMx still had a reasonably good performance with the lowest FDR and substantially better overall performance than glasso and k-glasso.

In Scenario IV (multiple graphical models), GGL, FGL, and MGGM had higher TPR compared to GGMx, however, at the price of higher FDR. Consequently, GGMx outperformed GGL, FGL, and MGGM in terms of FDR and the overall measure MCC.

In Scenario V, GGL and FGL were applied ignoring the continuous covariate whereas k-glasso was applied ignoring the discrete covariate. GGMx was able to simultaneously incorporate both continuous and discrete covariates in estimating graphs and therefore as expected it had the best performance compared to BGGM, glasso, k-glasso, GGL, and FGL in practically all measures.

In Scenario VI where the diagonal elements of **Ω**_*i*_ were not constrained to be constants, the results were consistent with those in Scenarios I-V. BGGM and glasso outperformed k-glasso overall but k-glasso was much better with respect to the selection of the edges that have substantial variability (measured by pTPR, pFDR, and pMCC). The proposed GGMx was clearly the best in both overall measures and partial measures. For example, GGMx had considerably higher MCC as well as pMCC than all the competing methods. In addition, we also evaluated the estimation accuracy of **Ω**_*i*_ by computing the mean squared error (MSE). We again focused on edges with true frequency of inclusion across observations between 0.1 and 0.9. The resulting MSE was 0.10, 1.24, 0.44, and 0.82 for GGMx, BGGM, glasso, and k-glasso, which demonstrated the capability of the proposed GGMx in capturing the heterogeneity in **Ω**_*i*_.

In Scenario VII, the main conclusion stays the same as in Scenario VI although k-glasso had significantly reduced FDR, however, at the price of significantly reduced TPR. GGMx, on the other hand, demonstrated its stable performance across all scenarios and all measures.

Lastly, we assessed the sensitivity of GGMx to the choice of all the hyperparameters (*a_τ_, b_τ_*) and (*μ_t_, σ_t_*). We picked Scenario VII and varied the hyperparameters in the following range, (*a_τ_, b_τ_*) ∈ {(10^−2^, 10^−2^), (10^−3^, 10^−3^), (10^−4^, 10^−4^)} and (*μ_t_, σ_t_*) ∈ {(1.0, 0.5), (1.0, 1.0), (1.5, 1.0)}^3^. The performance of GGMx with different hyperparameters is reported in Table 2, which shows GGMx is robust within the considered range.

## 7. Application in Multiple Myeloma

We present an application of GGMx in modeling transcriptomic regulation in multiple myeloma (MM) which is a late-stage malignancy of plasma cells. Recent research has shifted the focus from traditional “one size fits all” therapies to precision medicine strategies because MM is a highly heterogeneous genetic disease at an individual level (Hervé et al., 2011). To find better personalized treatment and more accurate prescriptive recommendations to MM patients, there needs to be a better understanding of the heterogeneity based on genomically defined pathways (Lohr et al., 2014). We use data generated by the Multiple Myeloma Research Consortium, a multi-institutional collaborative research effort collected data (among others) on gene expressions and clinical parameters from MM patients (Chapman et al., 2011).

**Figure 6:**
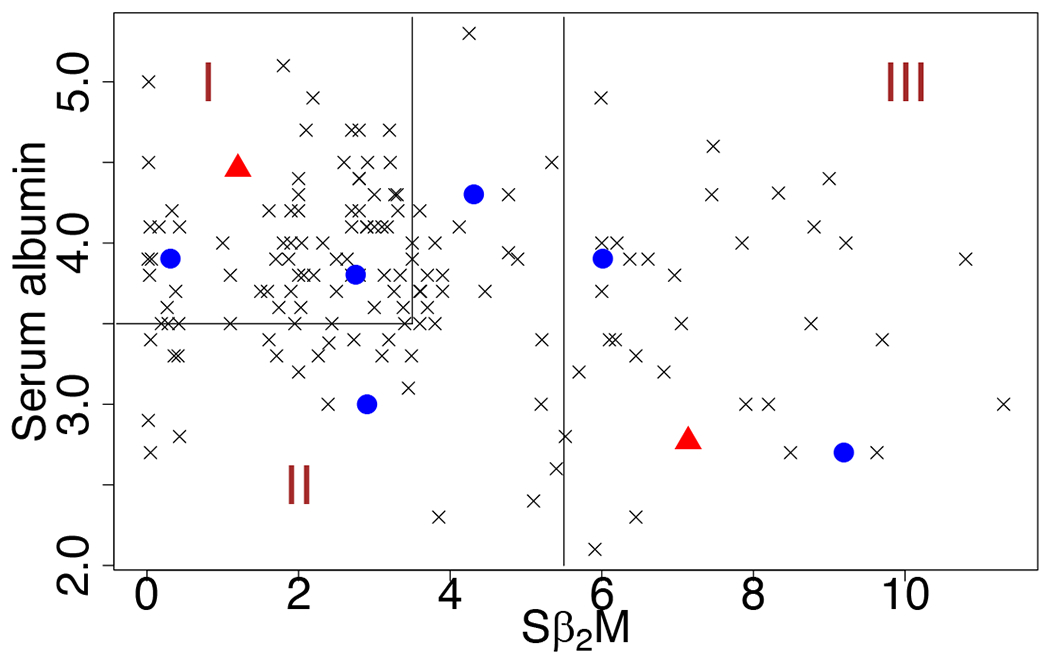
Observed prognostic factors are shown as crosses and dots. Dots are chosen as representative cases for network visualization in Figure 8. Triangles will be used to interpolate networks for unseen patients shown in Figure 9. The prognostic covariates space are partitioned into Stages I, II, and III, according to the International Staging System for multiple myeloma.

We focus our analyses on the genes mapped to one of the most important pathways in MM, NF-*κ*B signaling pathway. Activation of the NF-*κ*B pathway has been implicated in MM, but the genomic foundation of such activation is only partially understood (Demchenko et al., 2010; Roy et al., 2018). Clinical information includes measurements of two important prognostic factors, serum beta-2 microglobulin (S*β*_2_M) and serum albumin. The International Staging System (Greipp et al. 2005) uses these two prognostic factors to stage MM: stage I, S*β*_2_M < 3.5 mg/L and serum albumin ≥ 3.5 g/dL; stage II, neither stage I nor III; and stage III, S*β*_2_M ≥ 5.5 mg/L. The observed values of S*β*_2_M and serum albumin, and the staging partition are depicted in Figure 6. We use these two prognostic factors as covariates (*q* = 2).

The goal of this study was to infer subject-level gene expression networks whose structures are modified by the prognostic factors. After removing outliers and samples with missing gene expression or clinical information, we had *n* = 151 samples and *p* = 33 genes. We ran two separate MCMCs, each with 50,000 iterations, discarded the first 50% as burn-in and saved every 50th sample after burn-in. To check MCMC convergence, we calculated the potential scale reduction factor (PSRF, Gelman et al. 1992) for each entry in **Ω**_*i*_, *i* = 1, … , *n*. The median PSRF was 1.00 with interquartile range 0.01, which showed no lack of convergence. We then concatenated the two chains and all subsequent inference was based on the combined Monte Carlo samples. The probability cutoff *c* was chosen to control the posterior expected FDR at 1%.

### Population-level inference

**Figure 7:**
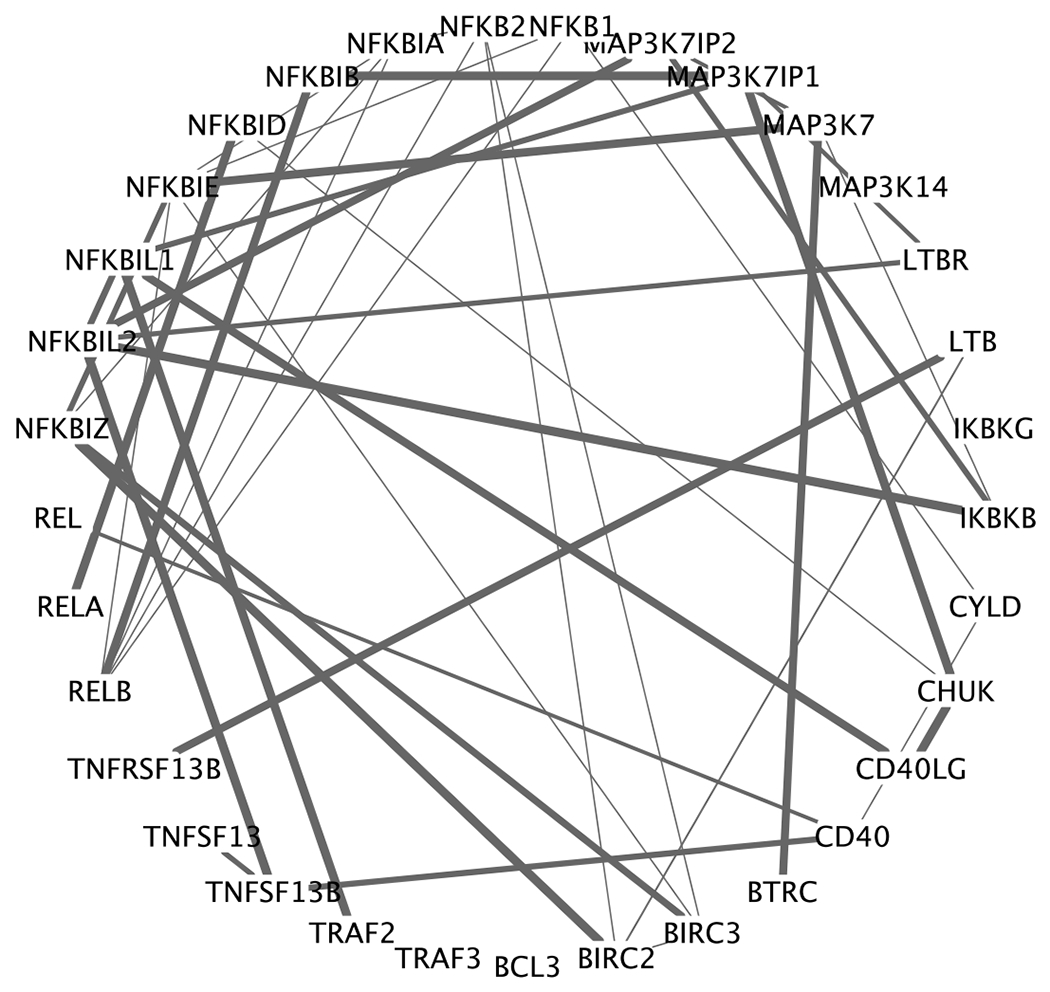
Population-level summary of gene expression network. The network is a union of all networks across subjects. The edge width is proportional to edge inclusion variability.

The estimated graphs had 30 edges per subject on average with minimum 20 edges (from a stage III patient) and maximum 37 edges (from a stage I patient). We summarized a population-level gene expression network *G* = (*V, E*) as the union of all networks across subjects $E=\cup_{i=1}^{n}E_{i}$. There were |*E*| = 42 edges in *G*. To visualize the graph variability, we computed the variance of edge inclusion. Specifically, let ***e**_jk_* = (*e*_1*jk*_ , … , *e*_*njk*_) be a binary vector such that *e_ijk_* = 1 if {*j, k*} ∈ *E*_*i*_. Then for edge {*j, k*}, the variance of edge inclusion was defined as the sample variance of ***e**_jk_*. The population-level network was reported in Figure 7, with the edge width proportional to edge inclusion variability. We found 14 out of 42 edges with variance greater than 0.2 (note the maximum variance is 0.25 for Bernoulli random variable). These 14 edges appeared in about 30%-70% of the patients. In line with our simulation studies, traditional GGMs are unlikely to accurately capture these differential edges.

### Subject-level inference

**Figure 8:**
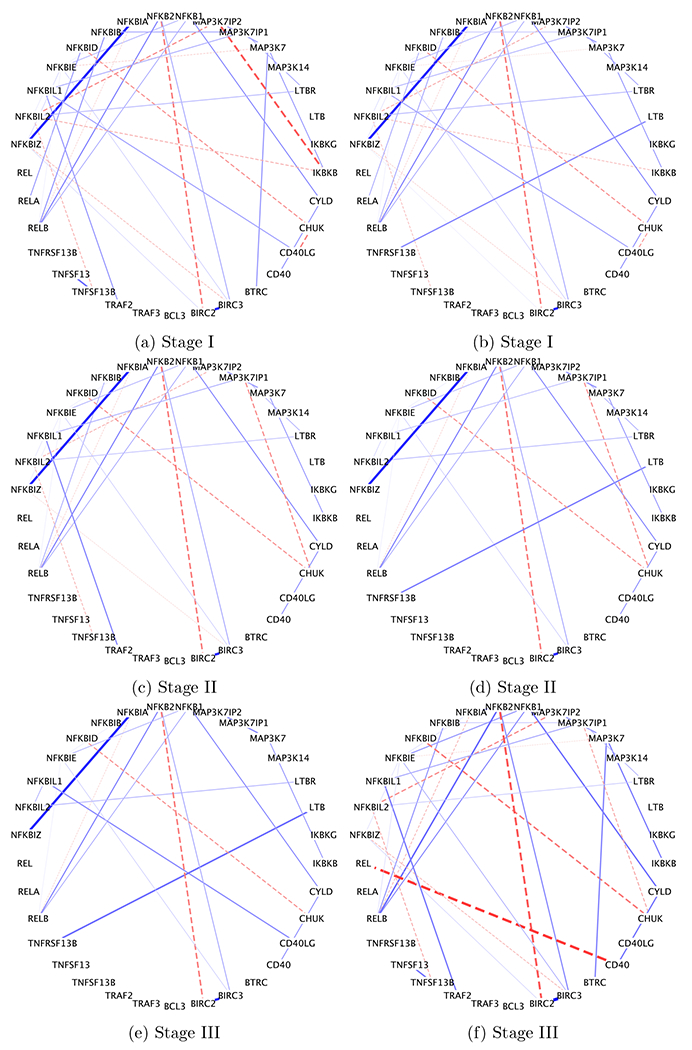
Subject-level networks for six representative patients, represented as dots in Figure 6. The edge width is proportional to the absolute value of partial correlation. The sign of partial correlation is represented by line type: + solid line and - dashed line.

Next, we focus on the subject-level inference. We chose 6 representative patients, 2 from each stage, to show their respective networks in Figure 8. The values of their prognostics factors are represented by the dots in Figure 6. We set the edge width proportional to the absolute value of partial correlation $\rho_{ijk}=-\frac{\omega_{ijk}}{\sqrt{\omega_{ijj}\omega_{ikk}}}$, and use solid lines to represent positive partial correlations and dashed lines negative partial correlations.

We highlight several interesting biological findings. RELB was found to be a highly connected gene across all patients (Figures 7 and 8). RELB is a core member of NF-*κ*B family. Hence it is not surprising that RELB played an import role in NF-*κ*B pathway. In fact, many MM patients have abnormal NF-*κ*B target gene expression, associated with genetic aberration of NFKB1 and NFKB2 (Annunziata et al., 2007). This further confirms our finding that RELB was consistently positively associated with NFKB1 and NFKB2. In addition, NFKBIA is an inhibitor of NF-*κ*B, which is consistent with our findings that NFKBIA was negatively associated with RELB across patients. It is also known that genes in the same family tend to be positively associated with each other. Our study found positive links, for example, BIRC2—BIRC3 and NFKBIA—NFKBIZ. As disease progresses, some paths get blocked and some new connections get acquired. Among others, the link between LTB and TNFRSF13B was found in stage III patients but not in stage I patients whereas the link between NFKBIL2 and MAP3K7IP2 was lost in stage III patients. While some of those links are well documented in the biological literature (Liu et al., 2017), their gain and loss mechanisms need further validation and investigation.

**Figure 9:**
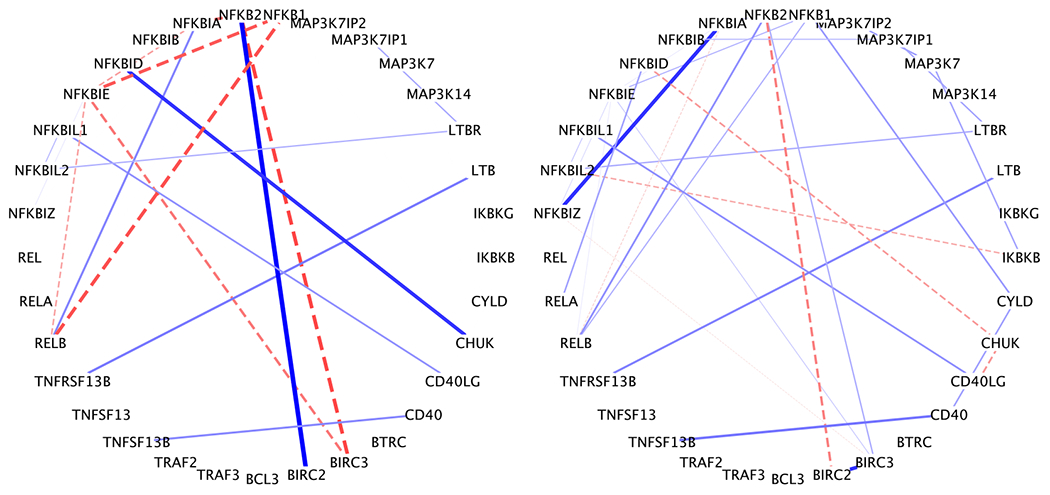
Network interpolation for two sets of unseen prognostic factors, represented as triangles in Figure 6.

Finally, as new patients come into the clinic, GGMx can be used to quickly predict the individualized gene network only based on the blood test results of S*β*_2_M and serum albumin without the costly and time-consuming whole genome sequencing. For illustration, we picked two sets of covariates that were unobserved in our collected data; they are represented by triangles in Figure 6. The estimated gene expression network of the two hypothetical patients are shown in Figure 9, which was enabled by the unique feature of graph interpolation of the proposed GGMx.

## 8. Discussion

In this article, we introduce a general regression framework for (undirected) Gaussian graphical models with covariates (GGMx). This generalization of regular GGM beyond i.i.d. data allows the graph structure and strength to change with covariates and is particularly challenging especially in the undirected graph context due to the positive definiteness constraint of a precision matrix. We have addressed this challenge through a novel prior that is theoretically connected to non-local priors for precision matrices, paired with a carefully designed MCMC algorithm for efficient posterior inference. GGMx includes at least five special cases including standard GGMs, group-specific GGMs, time-varying GGMs, covariate-dependent GGMs, and context-specific GGMs. We demonstrated the utility and robustness of GGMx through extensive simulations and an application in precision oncology. Our GGMx framework is broadly applicable to many other scientific domains of interest. For example, in brain functional magnetic resonance imaging data, GGMx can be used to study how brain connectivity networks change with covariates such as time and stimuli.

We remark that *covariance regression* (Hoff and Niu, 2012; Fox and Dunson, 2015) is a closely related model. It is, however, fundamentally different from the proposed GGMx in at least two ways. First, covariance regression assumes the covariance matrix rather than the precision matrix to be a function of ***x**_i_* which takes a specific form, $\Sigma_{i}=\Omega_{i}^{-1}=\Psi +\Lambda (x_{i})+\Lambda {\left(x_{i}\right)}^{T}$ for some PDM **Ψ** and matrix-value function **Λ**(***x**_i_*). Second, covariance regression assumes a dense **Σ**_*i*_ whereas GGMx allows **Ω**_*i*_ to be sparse and moreover, the sparsity pattern can change with covariates. Note that zeros in **Λ**(***x**_i_*) generally do not translate to zeros in **Σ**_*i*_ or **Ω**_*i*_. Therefore, it is not straightforward to extend the covariance regression framework to allow sparsity.

While our work is a useful first step for undirected graphical regression, there are several extensions and refinements possible. We have chosen the smooth covariate-dependent functions, *g_jk_*(·), to be linear for simplicity and parsimony. Same choice has been made by similar papers (Cheng et al., 2014). However, in general, it can be replaced by a nonlinear function. For example, letting $\tilde{x}$ denote some basis expansion of ***x*** such as splines and wavelets, we can model $g_{jk}(x)=\beta_{jk}^{T}\tilde{x}$ and the same inference procedure with linear functions applies. We plan to incorporate nonlinearity in our future work. Furthermore, we have worked with a moderate number of variables due to several reasons. First, the number of parameters that need to be estimated in GGMx is on the order of $\frac{p(p+1)(q+2)}{2}+\frac{p(p-1)}{2}$, which can be large even for a moderate number of variables and covariates. Second, from an application perspective, we focus on a specific signalling pathway in multiple myeloma, NF-*κ*B for deeper scientific interpretations. The small sample size (relative to the number of parameters) does not allow for reliable inferences for a much larger number of variables (e.g., the entire transcriptomic profile). Finally, the scalability of the proposed GGMx also limits the number of variables under consideration. The scalability can be potentially improved by adopting more efficient MCMC algorithms such as Metropolis-adjusted Langevin algorithm (Roberts et al., 1996) or Hamiltonian Monte Carlo (Duane et al., 1987). Both algorithms take advantage of gradient information of the target distribution. However, the hard thresholding function in (3) is discontinuous. This difficulty can be potentially overcome by considering a continuous relaxation of the hard thresholding function (Cai et al., 2018). Another potential solution is resorting to variational Bayes algorithms, which approximate the posterior distributions by simpler variational distributions through minimizing the Kullback–Leibler divergence between them. We hope to address the scalability issue in our future work.

## Figures and Tables

**Table 1:** Five special cases of GGMx.

Special cases of GGMx	Conditions	Mapping *X* ↦ Ω	
Standard GGM	***x**_i_* = 1	$g_{jk}\left(x_{i}\right)=\beta_{jk}$	$\omega_{ijk}=\beta_{jk}I\left(\left|\beta_{jk}\right|>t_{jk}\right)$
Group-specific GGM	***x**_i_* = *c*, *c* ∈ {1, … , *C*}	$g_{jk}\left(x_{i}\right)=\beta_{jkc}$	$\omega_{ijk}=\beta_{jkc}I\left(\left|\beta_{jkc}\right|>t_{jk}\right)$
Time-varying GGM	***x**_i_* = *x*, time x∈ℝ	$g_{jk}(x)=x\cdot \beta_{jk}$	$\omega_{ijk}=x\cdot \beta_{jk}I\left(\left|x\cdot \beta_{jk}\right|>t_{jk}\right)$
Covariate-dependent GGM	*t_jk_* = 0	$g_{jk}\left(x_{i}\right)=\beta_{jk}^{T}x_{i}$	$\omega_{ijk}=\beta_{jk}^{T}x_{i}$
Context-specific GGM	***x**_i_* = *y*_*i*1_	$g_{jk}\left(x_{i}\right)=y_{i1}\cdot \beta_{jk}$	$\omega_{ijk}=y_{i1}\cdot \beta_{jk}I\left(\left|y_{i1}\cdot \beta_{jk}\right|>t_{jk}\right)$

**Table 2:** Sensitivity Analysis. Operating characteristics for simulations under six alternative hyperparameter settings. The numbers are calculated on the basis of 50 repetitions; standard deviations are within parentheses. The first row shows the performance of GGMx in Scenario VII with default hyperparameter setting (*a_τ_, b_τ_*) = (10^−1^, 10^−1^) and (*μ_t_, σ_t_*) = (1, 0.2).

		TPR	FDR	MCC	pTPR	pFDR	pMCC
Default Parameter Setting		0.94 (0.04)	0.20 (0.12)	0.86 (0.07)	0.94 (0.04)	0.01 (0.01)	0.81 (0.09)
(*a_τ_, b_τ_*)	(10^−2^, 10^−2^)	0.93 (0.04)	0.15 (0.10)	0.89 (0.06)	0.93 (0.04)	0.01 (0.01)	0.81 (0.09)
	(10^−3^, 10^−3^)	0.93 (0.04)	0.10 (0.08)	0.91 (0.05)	0.93 (0.04)	0.01 (0.01)	0.80 (0.08)
	(10^−4^, 10^−4^)	0.93 (0.04)	0.07 (0.07)	0.93 (0.04)	0.93 (0.04)	0.01 (0.01)	0.80 (0.08)
(*μ_t_, σ_t_*)							
	(1.0, 0.5)	0.97 (0.03)	0.33 (0.12)	0.80 (0.08)	0.97 (0.03)	0.02 (0.02)	0.82 (0.07)
	(1.0, 1.0)	0.98 (0.02)	0.30 (0.13)	0.82 (0.08)	0.98 (0.02)	0.03 (0.02)	0.81 (0.08)
	(1.5, 1.0)	0.97 (0.03)	0.19 (0.11)	0.88 (0.07)	0.97 (0.03)	0.03 (0.02)	0.81 (0.08)

## Footnotes

1. This is a conceptual statement. Note that existing time-varying GGM methods typically assume graphs to vary non-linearly with time whereas this paper considers linearly-varying graphs.

2. Note that the probability cutoff *c* is different from the random threshold *t_jk_*. The random threshold is a model parameter used to induce sparsity whereas the probability cutoff is introduced to obtain posterior point estimate of graphs.

3. The resulting prior means (variances) of the minimum effect size *t_jk_* are 1.0 (0.22), 1.3 (0.63), and 1.6 (0.77).
